# Amyloid β peptides overexpression in retinal pigment epithelial cells via AAV-mediated gene transfer mimics AMD-like pathology in mice

**DOI:** 10.1038/s41598-017-03397-2

**Published:** 2017-06-12

**Authors:** Tuhina Prasad, Ping Zhu, Amrisha Verma, Paramita Chakrabarty, Awilda M. Rosario, Todd E. Golde, Qiuhong Li

**Affiliations:** 1 Department of Ophthalmology, University of Florida, Gainesville, Florida 32610 USA; 2 Department of Neuroscience, Center for Translational Research in Neurodegenerative disease and McKnight Brain Institute, University of Florida, Gainesville, Florida 32610 USA

## Abstract

Age-related macular degeneration (AMD) is a progressive retinal neurodegenerative disorder characterized by extracellular deposits known as drusen. A major constituent of drusen deposits are Alzheimer disease-associated amyloid β (Aβ) peptides. To understand the etiology of Aβ proteostasis in AMD, we delivered recombinant adeno-associated virus (AAV) encoding Aβ42 and Aβ40 peptides fused to BRI2 protein by intraocular injection in C57BL/6J mice. Endogenous protease cleavage of such constructs leads to production of secreted Aβ42 and Aβ40 respectively. We demonstrate that overexpression of secreted Aβ40 or Aβ42 resulted in dramatic induction of drusen-like deposits by 2 months’ post-injection. These drusen-like deposits were immunopositive for Aβ and complement proteins but did not stain for conventional amyloid dyes, such as Thioflavin S. Both injected cohorts showed gliosis and degenerative changes, though ERG responses were minimally affected. Intriguingly, simultaneous overexpression of BRI-Aβ40 or BRI-Aβ42 together resulted in dose-dependent and cumulative changes reminiscent of AMD type pathology - drusen-like deposits, severe reduction in ERG responses, photoreceptor cell loss and gliosis. Here, we have established a physiological model of Aβ containing deposits in wild-type mice that recapitulates major retinal pathophysiological features of AMD and will be instrumental in mechanistic understanding and development of therapeutic strategies against AMD.

## Introduction

Age-related macular degeneration (AMD) is a late-onset, progressive retinal neurodegenerative disease and the leading cause of severe and irreversible vision loss in the elderly in developed countries^1–5^. This disease primarily affects the macular region of human retina, which is crucial for central vision and perception of finer details, resulting in a progressive loss of high-resolution central vision in people afflicted with this disorder. The visual impairment associated with AMD occurs as a consequence of two different forms of the disease-dry AMD (geographical atrophy or nonexudative AMD), which is characterized by progressive retinal degeneration involving the photoreceptors and the retinal pigment epithelial (RPE) cells, or the more severe form- wet AMD (choroidal neovascularization or exudative AMD), which is characterized by the growth of new blood vessels from the choroid into the neural retina causing photoreceptor degeneration^2, 6, 7^. While wet AMD can be treated by laser photocoagulation, photodynamic therapy and intravitreal injections of anti-vascular endothelial growth factor (VEGF)-A and other angiogenic inhibitors, there is no effective treatment available currently for dry AMD^5, 8–10^. AMD is clinically characterized by the presence of extracellular deposits known as drusen located between the retinal pigment epithelium (RPE) and Bruch’s membrane (sub-RPE) in the macular region. Drusens are also observed in the normal aging eye, however the presence of large drusens in high numbers in the macular region has been identified as a significant risk factor for developing AMD^11–14^. The underlying pathogenesis of the disease is still not fully understood as it results from variable contributions of age, genetic predisposition, epigenetics and environmental factors. The complexity in the etiology of the disease has resulted in lack of effective therapies, preventative strategies and good animal models in which to study the disease^2, 15–18^.

Recent studies suggest that AMD shares many similarities with another age-related neurodegenerative disorder affecting the central nervous system (CNS): Alzheimer’s disease (AD), which could provide important insight into AMD pathogenesis and its treatment. Extracellular amyloid beta (Aβ) deposition, oxidative stress, and inflammation have all been implicated in both AD and AMD^14, 19, 20^. Although, the biological function of Aβ is not yet fully understood it is thought to have a role in synaptic physiology^21–23^. Aggregated Aβ42 and Aβ40 are major components of senile plaques in AD. Recent evidence shows that retinal ganglion cells (RGC) and the RPE also synthesize Aβ, which are secreted in the posterior eye^24^. Age associated changes in Aβ synthesis and degradation enzymes have been reported in the eye^25^ resulting in an elevated level of Aβ- rich extracellular deposits in both human and rodent aging retina^26, 27^, specifically associated with vesicular structures within the drusen^28–32^ and linked with key stages of AMD progression^26, 33^. Previous study has shown that Aβ-directed immunotherapy partially prevents retinal/RPE abnormalities in retinal degeneration mouse models^32^.Therefore, these secreted Aβ peptides are thought to be potential triggers for retinal degeneration in aged individuals and in AMD patients. Overall, these findings support the hypothesis that Aβ plays a crucial role in driving degenerative processes in the aging retina.

Most transgenic mouse models of AD use overexpression of mutant human amyloid precursor protein (APP) and presenilins (PS) to increase Aβ production and induce AD pathologies^34–36^. However, overexpression of APP results in not only increased production of both Aβ40 and Aβ42, the two most common peptides associated with AD pathogenesis, but also elevated levels of other APP fragments which can have neuroprotective, neurotoxic, or signaling functions that may complicate the interpretation of the results^37^. Increasing the expression of secreted human Aβ42 and Aβ40 via AAV mediated gene transfer of a fusion construct, between the type 2 transmembrane protein BRI2 (known to be involved in amyloid deposition in familial British dementia) and Aβ, into mouse hippocampus has shown to be effective in developing novel animal AD model^38–41^. Here, we used this method to selectively overexpress secreted Aβ42 and Aβ40 peptides in RPE of wild type mouse retina. We observed that intraocular delivery of AAV-BRI-Aβ resulted in AMD-like pathologies within eight weeks. Our findings provide definitive evidence that increased Aβ peptides in retina can trigger drusen-like extracellular deposits, retinal degeneration and loss of visual function seen in AMD patients. Overall, our findings reveal a novel, time-accelerated yet physiologically relevant model to study the molecular and cellular mechanisms underlying the toxic effect of specific Aβ-induced proteostasis dysfunction in AMD.

## Results

### Characterization of Aβ peptide expression following sub-retinal injection of AAV1-BRI, AAV1-BRI-Aβ40 and/or AAV1-BRI-Aβ42 vectors

**Figure 1:**
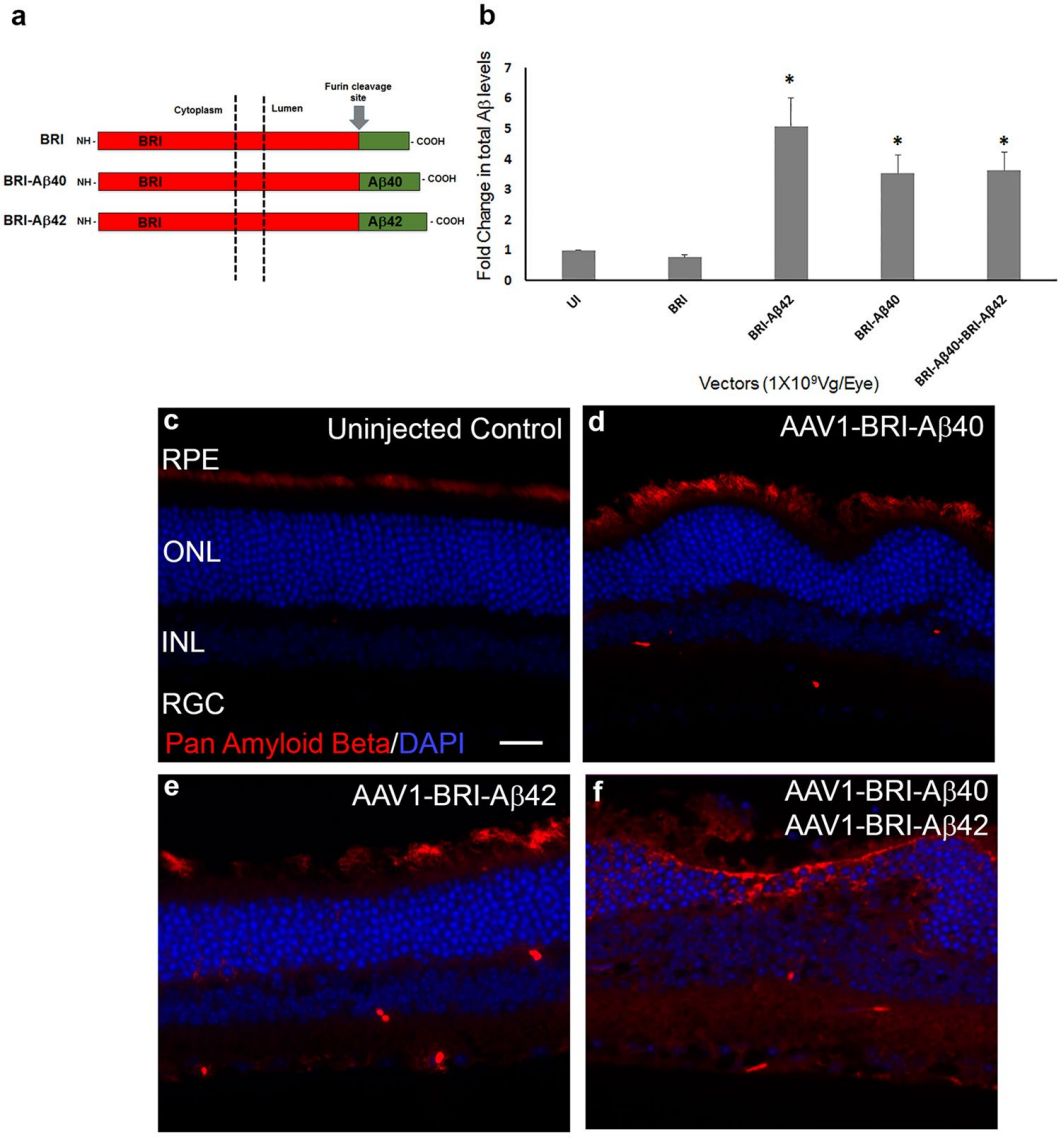
ELISA analysis and immunostaining to evaluate the expression of Aβ 8 weeks after sub-retinal injection of AAV1 vectors. (**a**) Schematic of the BRI-Aβ fusion constructs used to generate AAV1 vectors (modified from^41^) Control vector contains BRI protein that has been truncated at the protease site. (**b**) ELISA analysis for total Aβ showing a significant increase in the level of expression in the single vector injected and combination vector injected retinas when compared to age-matched un-injected (UI) and control vector AAV1-BRI injected retinas. An equal increase in level of Aβ expression in retinas receiving sub-retinal injection of AAV1-BRI-Aβ40, AAV1-BRI-Aβ42 and the combination of the two vectors AAV1-BRI-Aβ40+AAV1-BRI-Aβ42 is observed. n = 4/group, bars in this graph represent mean +/− SEM, Student’s t-test was performed to measure significance between the vector injected groups and the un-injected control group. *p < 0.05 is considered significant. (**c–f**) Aβ immunostaining of retinal sections from each experimental group shows that human Aβ is expressed only in eyes that received sub-retinal injection of AAV1-BRI-Aβ40, AAV1-BRI-Aβ42 and AAV1-BRI-Aβ40+AAV1-BRI-Aβ42. Scale Bar = 20 μm. RPE: retinal pigment epithelium﻿; ﻿ONL: outer nuclear layer; INL: inner nuclear layer; IPL: inner plexiform layer; RGC: retinal ganglion cells.

The detailed description of generating AAV construct expressing the cDNA of fusion proteins between type 2 transmembrane protein BRI2 and Aβ40 or Aβ42 was reported previously^38, 39^. Briefly, AAV1-BRI - a truncated form of BRI2 protein with a mutation at the protease site (that blocks cleavage of the ABri peptide from BRI protein^39^) was used as a control vector. The BRI-Aβ fusion construct was generated by fusing the Aβ40 or Aβ42 to the C-terminus of the BRI protein at the furin cleavage site. Cleavage by furin or furin like proteases results in the release of Aβ into the lumen or extracellular space (Fig. 1a).

The level of total Aβ in the retinal tissue was measured using a commercial ELISA kit eight weeks after the sub-retinal injection of AAV1 vectors encoding BRI (control vector), BRI-Aβ40, BRI-Aβ42, and co-injection of both AAV1-BRI-Aβ40 and AAV1-BRI-Aβ42 at the same final viral dose. The total Aβ level in the group injected with the control vector AAV1-BRI was not different from un-injected control eyes (Fig. 1). Whereas, a 3.5 to 4.5 fold increase in the level of total Aβ expression was observed in the groups receiving AAV1-BRI-Aβ40, AAV1- BRI-Aβ42 or a combination of AAV1-BRI-Aβ40 and AAV1-BRI-Aβ42 when compared with AAV1-BRI injected or un-injected eyes. The increase in total level of Aβ in the single vector injected groups did not show a significant difference when compared to the combination vector injected group, thus providing evidence that an equal amount of total vector was injected and expressed in all the experimental groups (Fig. 1b). Immunostaining with an Aβ antibody that specifically detects the human Aβ peptides showed that sub-retinal injection of AAV1 vectors encoding BRI-Aβ40 and/or BRI-Aβ42 resulted in transgene expression mostly in the outer retina confirming that these peptides are expressed from the RPE cells and secreted into the sub-retinal space and neighboring retinal cell types (Fig. 1c–e). In the un-injected and AAV1-BRI injected eyes a very faint staining is observed in the outer segment of the photoreceptor and RPE layer (Fig. 1c). A similar faint staining was also observed in the secondary antibody only and isotype control slides (Supplementary Fig. S1) suggesting that it was due to background rather than cross-reactivity to endogenous mouse peptides. However, interestingly these Aβ positive regions did not stain positive with Thioflavin S, a conventional amyloid dye that is used to stain amyloid plaques in AD brain (Supplementary Fig. S2).

### Aβ peptides induced retinal pathophysiology evaluated by fundus and OCT

**Figure 2:**
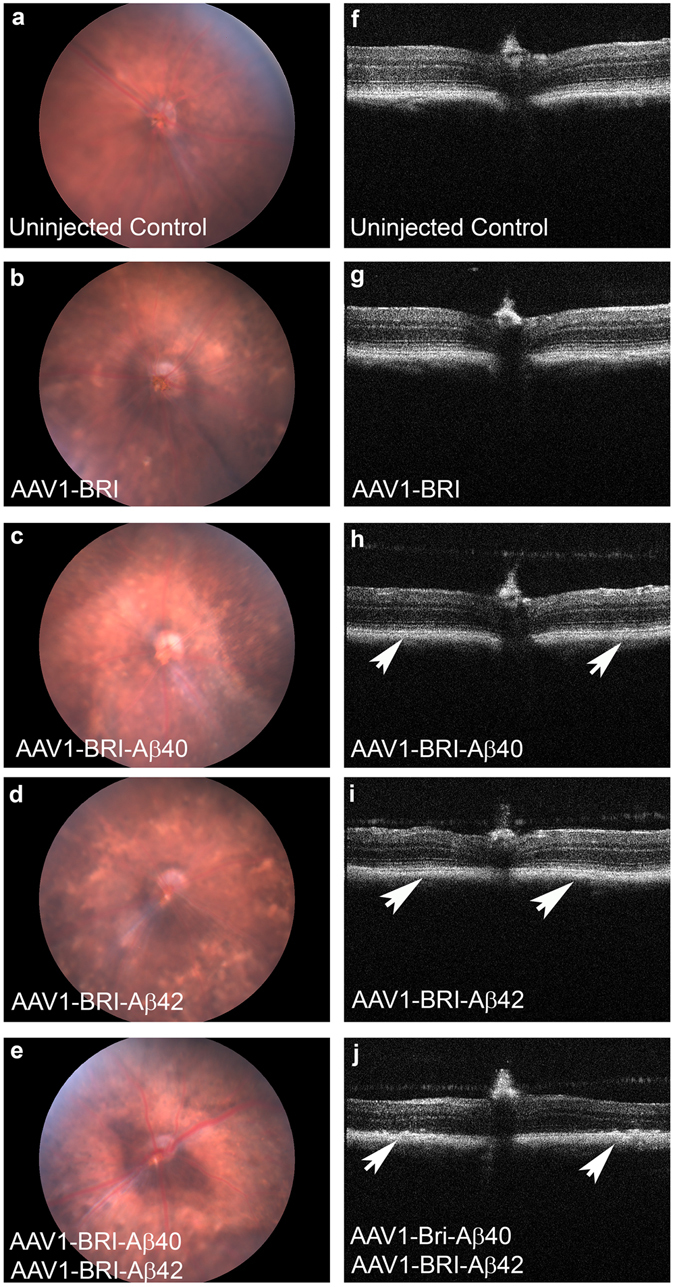
Fundus and OCT images of mouse eyes 6 weeks after sub-retinal injection. (**a–e**) Fundus images of (**a**) un-injected eye, (**b**) injected with AAV1-BRI (**c**) AAV1-BRI-Aβ40 (**d**) AAV1-BRI-Aβ42 and (**e**) AAV1-BRI-Aβ40+AAV1-BRI-Aβ42 injected eyes. (**f–j**) OCT images of (**f**) un-injected eye, (**g**) injected with AAV1-BRI, (**h**) AAV1-BRI-Aβ40, (**i**) AAV1-BRI-Aβ42 and (**j**) AAV1-BRI-Aβ40+AAV1-BRI-Aβ42. Arrows mark areas of drusen-like deposits.

*In vivo* retinal morphology was assessed by non-invasive fundus and OCT imaging every 2 weeks until 8 weeks after sub-retinal injection when the mice were sacrificed for end-point assays. Fundus and OCT images show an increasing number of drusen-like deposits in the sub-retinal space in eyes injected with AAV1-BRI-Aβ42, AAV1-BRI-Aβ40 and a combination of AAV1-BRI-Aβ42+AAV1-BRI-Aβ40 when compared to AAV1-BRI (control vector) injected or un-injected eyes (Fig. 2). The number and size of drusen-like deposits were more prominent in eyes with the combination vector injection. The presence of drusen-like deposits became evident as early as three weeks after the vector injection and progressively increased in number and size with time.

### Overexpression of Aβ peptides impaired visual function evaluated by ERG

**Figure 3:**
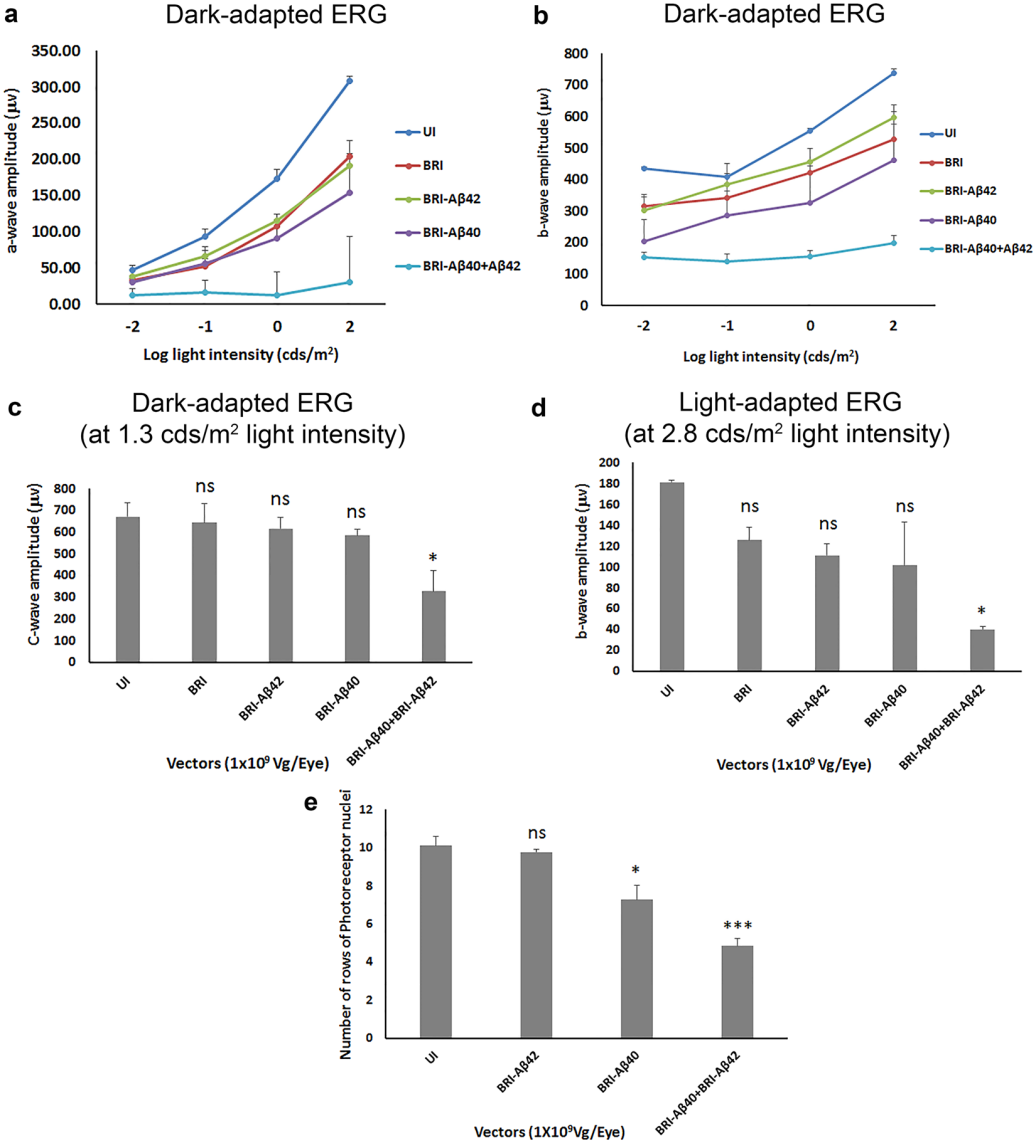
Quantification of ERG a-, b- and c- wave amplitude 8 weeks after sub-retinal injection. (**a**) Mean dark-adapted ERG a-wave amplitude for the different experimental groups at different light intensities. (**b**) Mean dark-adapted ERG b-wave amplitude for different experimental groups at different light intensities. (**c**) Mean dark-adapted ERG c-wave amplitude at ~1.3 cds/m^2^ light intensity. (**d**) Mean light-adapted ERG b-wave amplitude at ~2.7 cds/m^2^ light intensity. (**e**) Quantification of number of rows of photoreceptor nuclei in retinal sections from each experimental group. n = 6/group, points/bars in graph represent mean +/− SEM. Student’s t-test was performed to measure significance between the vector injected groups and the un-injected control group. *p < 0.05 is considered significant, ***p < 0.001 is considered highly significant, ns = not significant. Vg: vector genome.

Visual function was evaluated by analysis of full field scotopic and photopic ERG at 6 and 8 weeks after injection. Significant reduction in a-wave, b-wave and c-wave amplitudes of scotopic ERG in AAV1-BRI-Aβ40+AAV1-BRI-Aβ42 injected eyes were observed at both 6 weeks (data not shown) and 8 weeks after vector injection compared to the age-matched un-injected and AAV1-BRI injected eyes. In addition, a slight reduction in the scotopic ERG amplitudes in AAV1-BRI-Aβ42 and AAV1-BRI-Aβ40 single vector injected eyes were also observed. However, this reduction did not prove to be statistically significant when compared to age-matched un-injected and AAV1-BRI injected eyes (Fig. 3a–c). Similar trends were observed in the photopic b-wave amplitude wherein the decrease in the ERG amplitude is statistically significant only in eyes that received sub-retinal injection of AAV1-BRI-Aβ40+AAV1-BRI-Aβ42 compared to the un-injected and AAV1-BRI injected eyes (Fig. 3d). Reduced ERG responses in eyes co-injected with AAV1-BRI-Aβ40 and AAV1-BRI-Aβ42 vectors correlated with significant loss of photoreceptors (Fig. 3e). Interestingly eyes injected with AAV1-BRI-Aβ40 also showed significant loss of photoreceptor compared to un-injected or AAV1-BRI injected eyes (Fig. 3e) even though the ERG response was not significantly reduced.

### Aβ peptides induced retinal pathophysiology evaluated by histology

**Figure 4:**
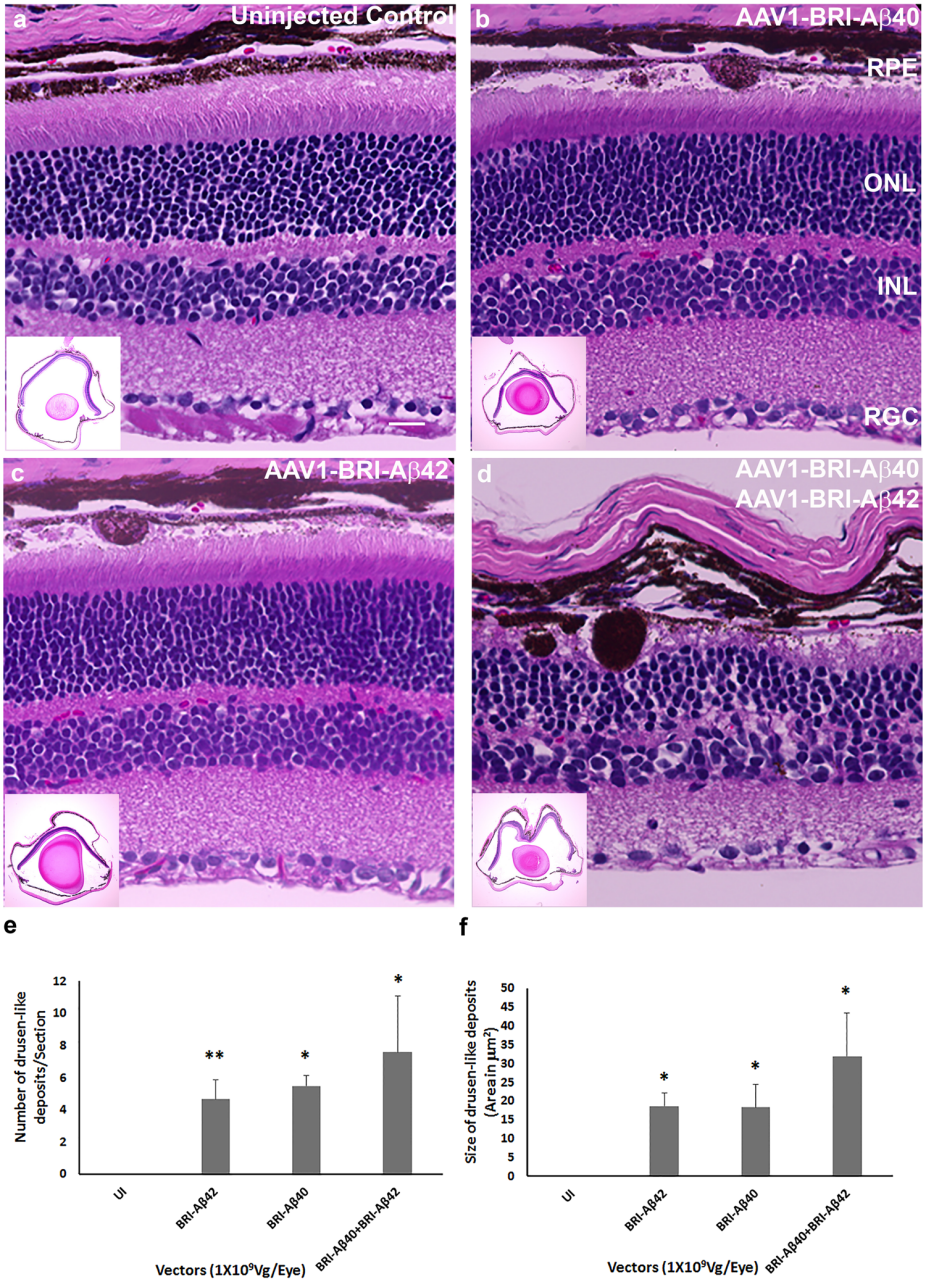
H&E staining to detect retinal morphology 8 weeks after sub-retinal injection of AAV1 vectors. Representative images of H&E staining at 400X magnification and at low 4X magnification showing whole retina in inset (**a**) un-injected eye, (**b**) AAV1-BRI-Aβ40, (**c**) AAV1-BRI-Aβ42 and (**d**) AAV1-BRI- Aβ40+AAV1-BRI-Aβ42 injected eyes. Scale bar: (**a–d**) = 20 μm. RPE: retinal pigment epithelium; ONL: outer nuclear layer; INL: inner nuclear layer; IPL: inner plexiform layer; RGC: retinal ganglion cells. (**e**) Quantification of number of drusen-like deposits in retinal sections from each experimental group. (**f**) Quantification of total area of drusen-like deposits in retinal sections from each experimental group. n = 10/group, bars in this graph represent mean +/− SEM. Student’s t-test was performed to measure significance between the vector injected groups and the un-injected control group. *p < 0.05 is considered significant, **p < 0.01 is considered highly significant.

H&E staining of paraffin sections were used to evaluate the retinal morphology 8 weeks after sub-retinal injection. Drusen-like deposits were evident in the sub-retinal space in all eyes injected with AAV1-BRI-Aβ40, AAV1-BRI-Aβ42 and AAV1-BRI-Aβ40+AAV1-BRI-Aβ42 but absent in the un-injected and AAV1-BRI injected eyes (Fig. 4). In single vector injected eyes, smaller and fewer number of drusen-like deposits were visible and there was no apparent damage to the photoreceptor layer in the AAV1-BRI-Aβ42 injected eyes (Fig. 4b and c). In the eyes injected with the combination vector, the number and size of drusen-like deposits were significantly more profound and additionally were associated with severe damage to photoreceptors and the RPE layer (Fig. 4d–f). No drusen-like deposits or damage to photoreceptor and RPE layer were seen in the AAV1-BRI injected eyes.

### Overexpression of Aβ peptides resulted in increased apoptosis, gliosis and inflammation

**Figure 5:**
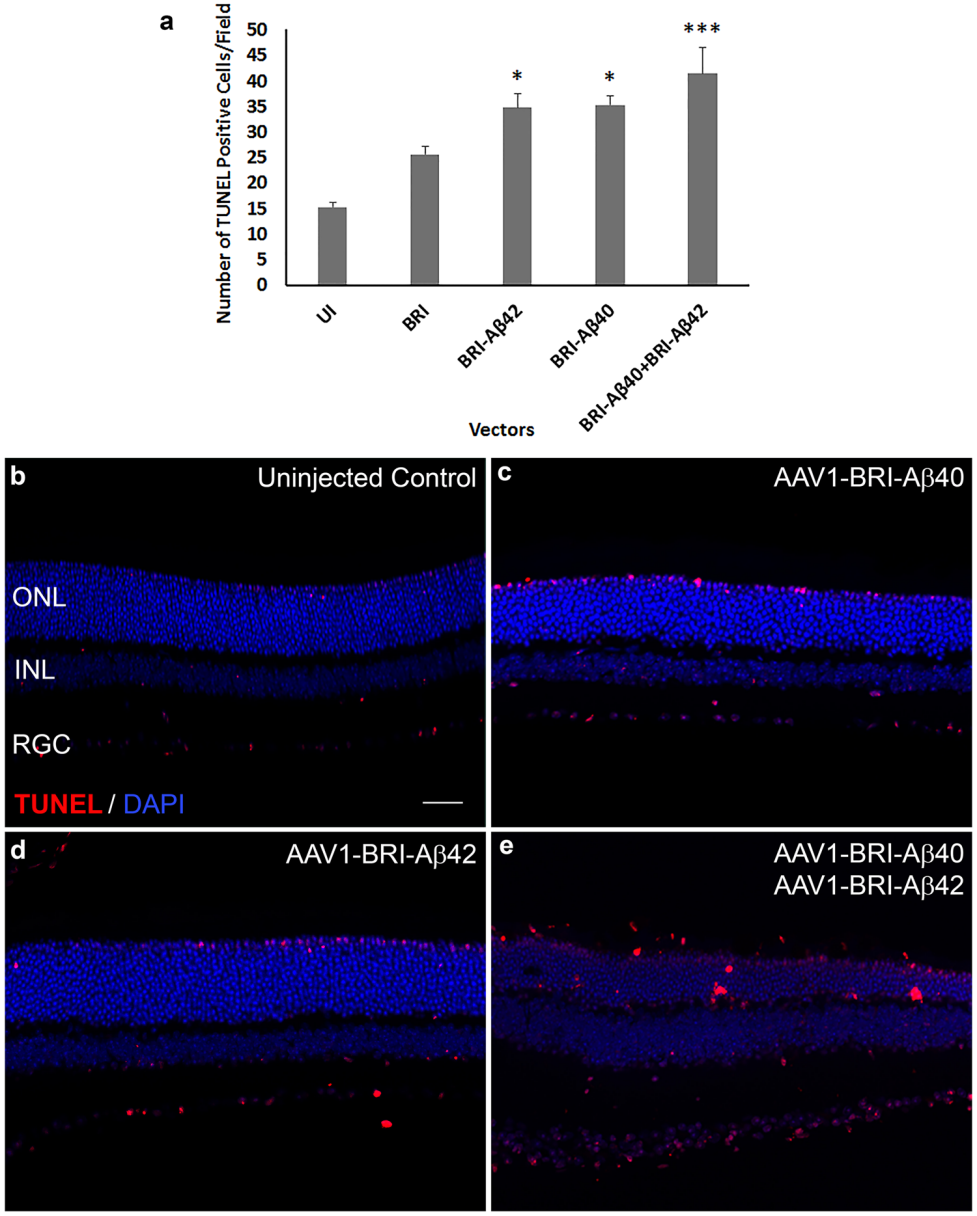
TUNEL labeling to measure cell death 8 weeks after sub-retinal injection. (**a**) Quantification of TUNEL positive cells in AAV1-BRI-Aβ vector injected eyes. (**b–e**) Representative images of TUNEL labeling to identify apoptotic cells at 400X magnification in (**b**) un-injected eye (**c**) AAV1-BRI-Aβ40 (**d**) AAV1-BRI-Aβ42 and (**e**) AAV1-BRI-Aβ40+AAV1-BRI-Aβ42 injected eyes. Scale bar: (**b–e**) = 20 μm. ONL: outer nuclear layer; INL: inner nuclear layer; IPL: inner plexiform layer; RGC: retinal ganglion cells. *p < 0.05 is considered significant, ***p < 0.001 is considered highly significant.

**Figure 6:**
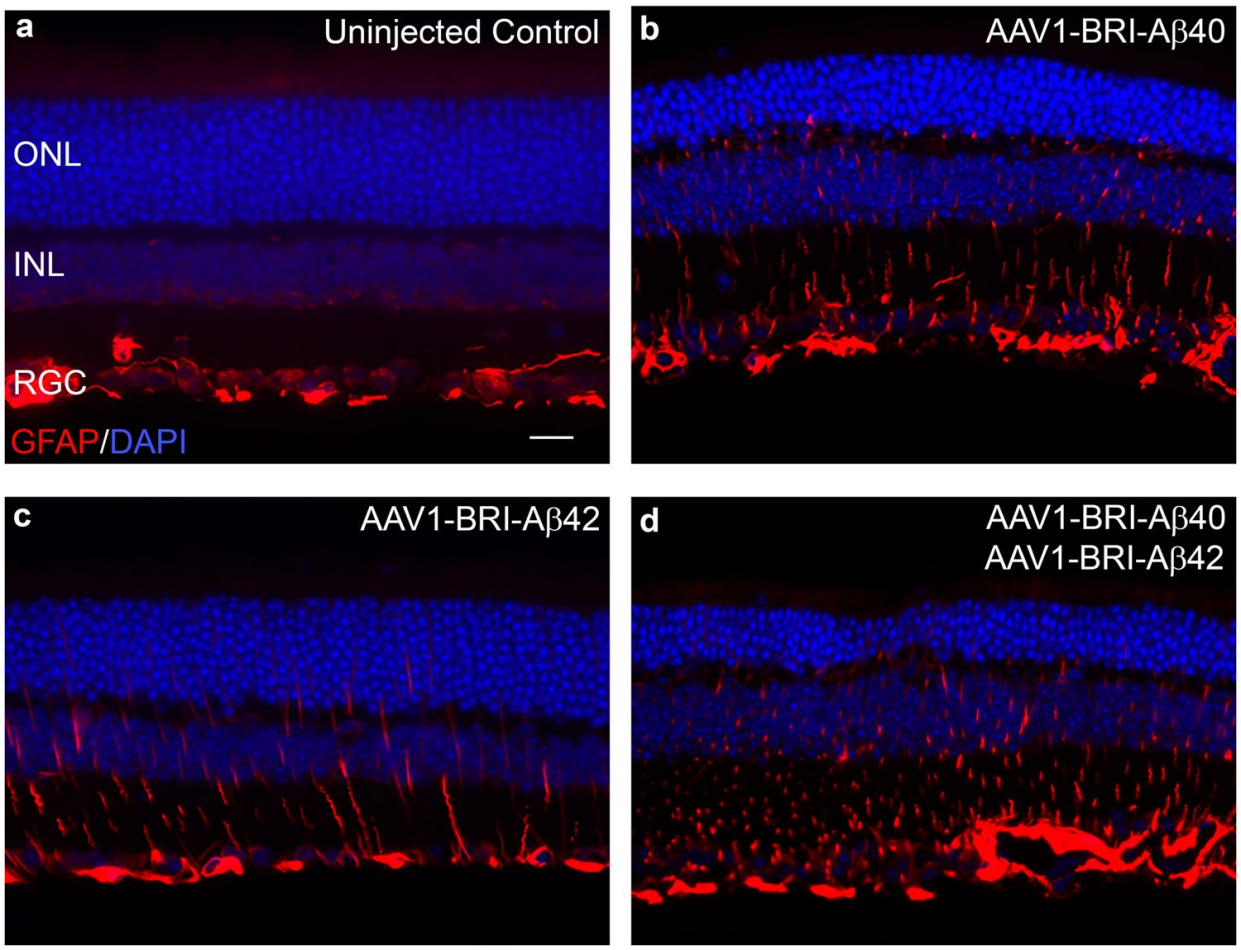
GFAP immunostaining to measure retinal gliosis 8 weeks after sub-retinal injection. GFAP staining was mainly confined to astrocytes and ganglion cell layer at the inner limiting membrane in the un-injected control mice. In AAV1-BRI-Aβ injected mice increased GFAP expression along the Müller glial cell process is observed extending towards the outer limiting membrane (**a–d**). GFAP expression in (**a**) Un-injected eye, (**b**) AAV1-BRI-Aβ40, (**c**) AAV1-BRI-Aβ42, and (**d**) AAV1-BRI-Aβ40+AAV1-BRI-Aβ42 injected eyes. Scale bar: (**a–d**) = 20 μm. ONL: outer nuclear layer; INL: inner nuclear layer; IPL: inner plexiform layer; RGC: retinal ganglion cells.

**Figure 7:**
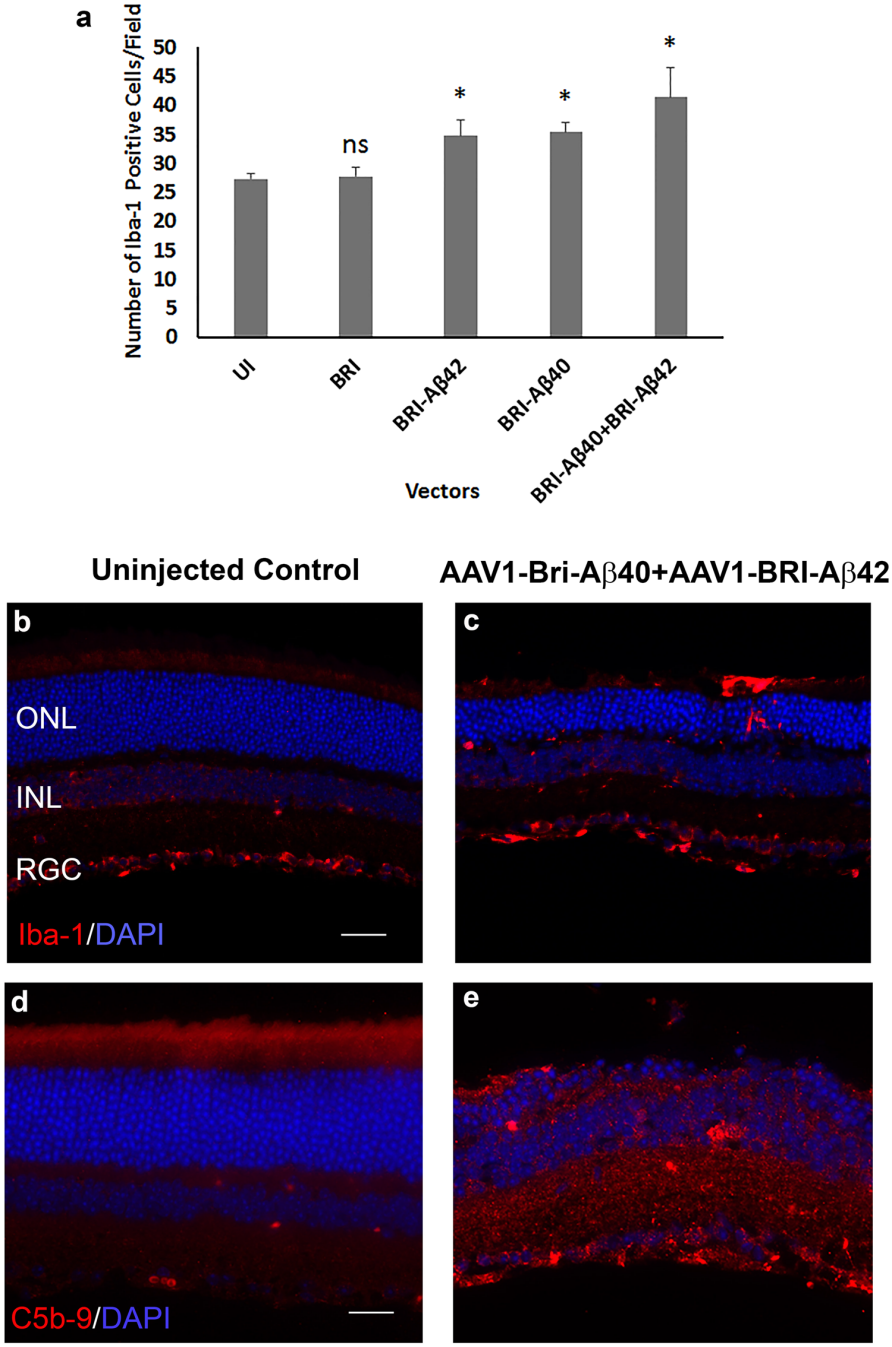
Iba-1 labeling of microglia and C5b9 labeling the complement activation. (**a**) Quantification of number of Iba-1 positive cells in AAV1-BRI-Aβ vector injected eyes. (**b,c**) Representative images of Iba-1 labeling in un-injected control eyes and the combination vector AAV1-BRI-Aβ40+AAV1-BRI-42 injected eye. There is an increase in the total number of Iba-1 positive cells and the number of activated microglial cells migrating to the outer retina in vector injected eyes compared to the un-injected and control vector injected eyes. Scale bar: (**b,c**) = 20 μm. (**d,e**) Representative images of C5b9 labeling in un-injected control eyes and the combination vector AAV1-BRI-Aβ40+AAV1-BRI-Aβ42 injected eye. Scale bar: (**d,e**) = 10 μm. ONL: outer nuclear layer; INL: inner nuclear layer; IPL: inner plexiform layer; RGC: retinal ganglion cells. *p < 0.05 is considered significant, ns = not significant.

Retinal cell death was evaluated by TUNEL labeling in paraffin sections from different experimental groups. Sub-retinal injection of AAV1-BRI-Aβ42, AAV1-BRI-Aβ40 and AAV1-BRI-Aβ40+AAV1-BRI-Aβ42 resulted in increased numbers of positively labeled cells mainly in the photoreceptor layer (Fig. 5) eight weeks post sub-retinal injection. Additionally, retinal gliosis was evaluated by immuno-staining for glial fibrillary acidic protein (GFAP) and Iba1. GFAP positive staining is confined in astrocytes in normal un-injected eye (Fig. 6a), but highly elevated in Müller glial cells in eyes that received injection of AAV1-BRI-Aβ40, AAV1-BRI-Aβ42, or combination of both vectors AAV1-BRI-Aβ40+AAV1-BRI-Aβ42 compared to the AAV1-BRI injected or un-injected control eyes (Fig. 6). Staining for Iba-1 also revealed that sub-retinal injection of AAV1-BRI-Aβ40+AAV1-BRI-Aβ42 vectors resulted in increased levels of microglia and macrophages compared to the AAV1-BRI injected or un-injected control eyes. Double immunostaining with Iba-1 and pan Aβ showed that although there is an increase in Iba-1 signal in the combination vector injected mice retina there is no co-localization between the two signals thereby excluding the possibility of any cross-reactivity with the microglial Fc receptors (Supplementary Fig. S3). Immunostaining for C5b9, a marker for activated complement cascade, showed that the drusen-like deposits observed in AAV1-Bri-Aβ40+AAV1-BRI-Aβ42 vectors injected eyes were positive for C5b9 (Fig. 7).

### Aβ peptide induced increased expression of pro-inflammatory cytokines

**Figure 8:**
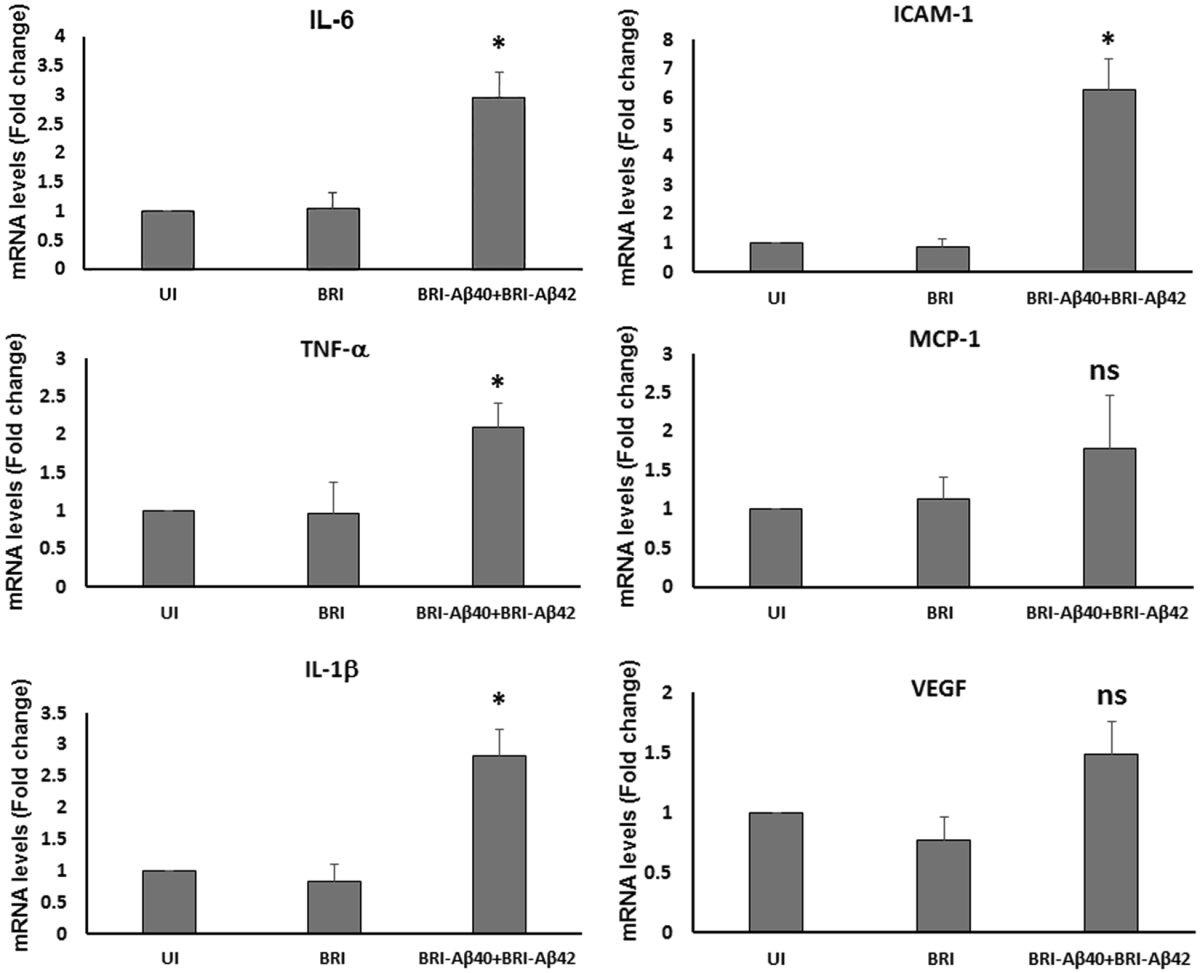
Real-time reverse transcriptase (RT)-PCR analysis of retinal mRNA levels of pro-inflammatory cytokines and VEGF. Value on Y-axis represents fold difference compared to age-matched wild-type un-injected retinal samples for each gene. UI: un-injected; BRI: AAV1-BRI and BRI-Aβ40+BRI- Aβ42: combination vector AAV1-BRI-Aβ40+AAV1-BRI-Aβ42 injected retinal samples. Data is expressed as mean+SD, n = 3–4/group. Student’s t-test was performed to measure significance between the combination vector injected group and the un-injected control group. *p < 0.05 is considered significant, ns = not significant.

Real-time RT-PCR was performed to evaluate the effect of Aβ peptides on the expression of pro-inflammatory cytokines and VEGF in the retina. In the combination vector injected retinas (AAV1-BRI-Aβ40+AAV1-BRI-Aβ42) a significant increase in the mRNA levels of Interleukin -6 (IL6), Tumor necrosis factor-α (TNF-α), Interleukin 1β (IL-1β) and Intercellular adhesion molecule 1 (ICAM1) were observed when compared to the age-matched un-injected retinas (Fig. 8). An increase was also observed in the level of monocyte chemoattractant protein-1 (MCP-1) but this increase was not statistically significant. There was no significant difference in the mRNA levels of the inflammatory cytokines in the AAV1-BRI injected retinas when compared to the un-injected retinas. Also, there was no significant increase in the level of VEGF in the injected retinas when compared to the age-matched un-injected retinas or the AAV1-BRI injected retinas thus reducing the possibility of Aβ peptides induced neo- vascularization or development of wet AMD symptoms in this model at this time point (Fig. 8).

### Combination of Aβ42 and Aβ40 peptides is more toxic than single Aβ peptide even at lower doses

**Figure 9:**
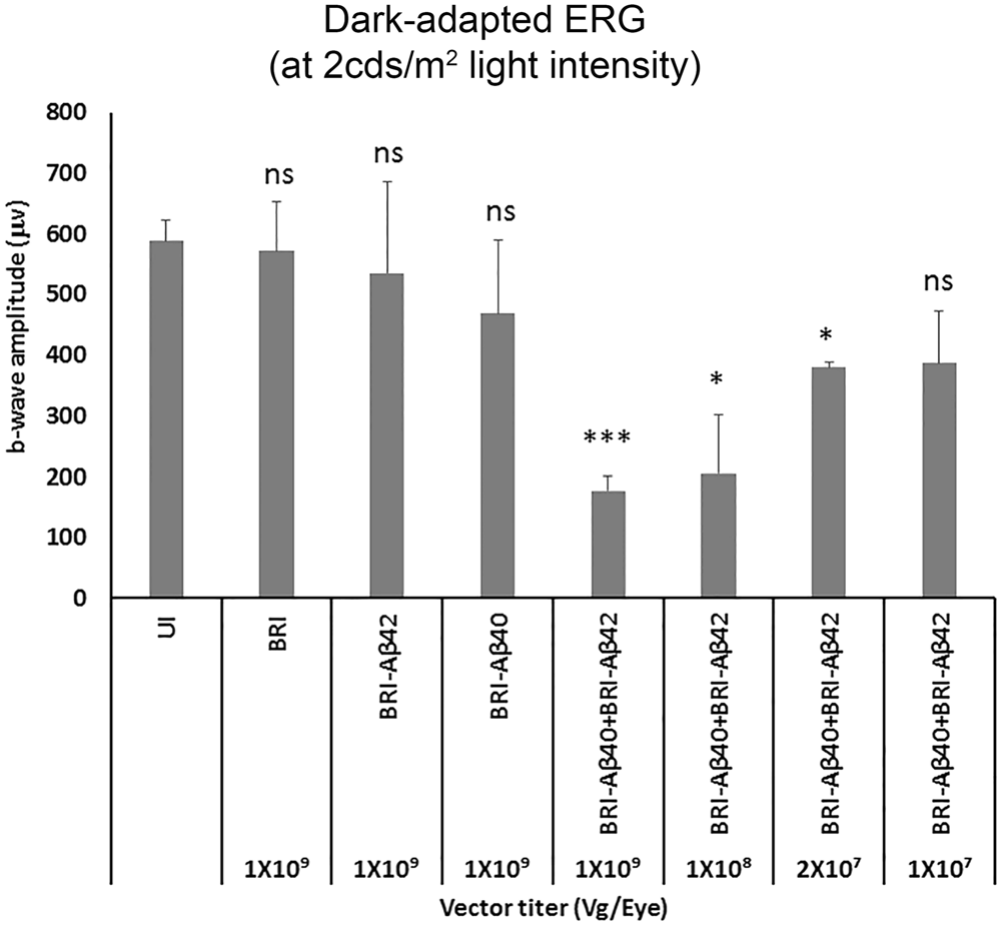
Dose-dependent effects of AAV1-BRI-Aβ40+AAV1-BRI-Aβ42 on visual function evaluated by ERG Graph shows the mean dark-adapted ERG b-wave amplitudes at ~2cds/m^2^ light intensity in different groups injected with either single vector or combination vector at different titers. n = 5/group, bars in this graph show mean +/− SEM. Student’s t-test was done to measure significance between the vector injected groups and the un-injected control group. *p < 0.05 is considered significant, ***p < 0.001 is considered highly significant, ns = not significant.

The animals injected with combination of AAV1-BRI-Aβ40+AAV1-BRI-Aβ42 vectors showed more severe loss in visual function when compared to single vector injected animals at the same dose. To test whether this effect was dose dependent, lower doses of the combination vector were injected and visual function was evaluated by full field scotopic and photopic ERG at eight weeks after injection. Both 10- and 50- fold lower titers (1 × 10^8^ vg/eye and 2 × 10^7^ vg/eye respectively) of the combination vector resulted in a statistically significant decrease in amplitudes of ERG a-wave, b-wave and c-wave when compared to single vector injections at full dose (1 × 10^9^ vg/eye) (Fig. 9).

## Discussion

Our present study shows that intra-ocular co-injection of AAV1-BRI-Aβ40 and AAV1-BRI-Aβ42, which results in increased secretion of Aβ peptides in the sub-retinal region, generates a physiologically relevant model for dry AMD in a significantly short time span of about 8 weeks. These mice develop drusen-like deposits that are immune-positive for Aβ in the sub-retinal region. Additionally the drusen-like deposits is accompanied with degeneration of surrounding RPE and photoreceptor cells, gliosis, activation of microglial cells and a significant decrease in ERG wave amplitude.

Clinical and experimental evidences have implicated Aβ40/42, peptides that are highly prone to aggregation and known to cause AD, as a trigger for drusen formation and development of AMD^14, 30, 42–46^. Under physiological condition Aβ peptides are produced by enzymatic processing of APP and is degraded by peptidases like neprilysin^25^. However, increasing the expression level of APP (which degrades into number of peptides some of which have neuroprotective or neuro signaling functions) or inhibiting neprilysin (which acts on a broad range of substrates with varied functions) would result in multiple effects in addition to increasing the level of Aβ peptides thereby greatly complicating the interpretation of results. To circumvent these issues, we developed an experimental approach that allowed precise manipulation of specific Aβ peptide expression level in mouse eyes. Here, we increase the expression level of secreted human Aβ40 and Aβ42 peptides in mouse retina via sub-retinal injection of AAV1-BRI-Aβ40 or/and AAV1-BRI-Aβ42. 8 weeks’ post-injection the combination vector injected mice eye show a clear increase in pigmentation associated with an increase in the number of drusen-like deposits. We hypothesize that the increase in secreted Aβ peptide acts as a trigger that initiates inflammatory response, activation of the complement cascade, degeneration of RPE and photoreceptor cells and deposition of extracellular material that goes on to form the drusen-like structure. Further studies to analyze the composition of these drusen-like structures developed in this model will be required.

The AAV1 serotype for gene transduction and the sub-retinal route for intra-ocular injection used in this study where chosen specifically because it allowed for the primary transduction of RPE cells, which is one of the major cell types implicated in AMD pathogenesis. Mouse eyes injected with AAV1-BRI-Aβ40 and/or AAV1-BRI-Aβ42 showed a build-up of Aβ- rich extracellular deposits in the retina, primarily along bruch’s membrane and the outer segments of photoreceptors. A similar pattern of Aβ expression is also observed in human AMD patient’s retina (Supplementary Fig. 4) and in normal aged mice and human retina. A subsequent decrease in the number of photoreceptor nuclear layers is observed in the AAV1-BRI-Aβ40 and the combination of AAV1-BRI-Aβ40+AAV1-BRI-Aβ42 injected mouse eyes, but not in the AAV1-BRI-Aβ42 injected eyes. Additionally, there is a significant reduction in ERG response only in eyes co-injected with equal titers of AAV1-BRI-Aβ40 and AAV1-BRI-Aβ42. Eyes injected singly with either AAV1-BRI-Aβ40 or AAV1-BRI-Aβ42 did not show a significant decrease in the ERG response suggesting a higher effectiveness of the combination vector in developing AMD like symptoms.

Aβ activated inflammatory and oxidative stress pathways in the eye are strongly implicated in progression of AMD^47^, also the formation of drusen in AMD is known to be associated with activation of the complement cascade^45, 48^. In our present study, we observe a colocalization of the activated complement marker C5b9/MAC at the sub-retinal drusen- like deposits, microglial activation, and an increase in the level of pro-inflammatory cytokines in both individual AAV1-BRI-Aβ40 and AAV1-BRI-Aβ42 injected eyes and in the dual AAV1-BRI-Aβ40+AAV1-BRI-Aβ42 vector injected eyes. In contrast to earlier reports^40, 47, 49, 50^, our present study suggests that Aβ40 seems to be more toxic to photoreceptor than Aβ42. Interestingly, the overexpression of a combination of Aβ40+Aβ42 caused more severe retinal pathophysiology even at a 50-fold lower dose when compared to overexpression of either Aβ40 or Aβ42 individually suggesting that it is possible that the two peptides interact with each other to cause a more pathogenic effect. An earlier study shows that an equal level of Aβ40+Aβ42 peptide together proved to be more toxic to cultured neuronal cell when compared to Aβ40 or Aβ42 peptide alone^51^. The ratio of the two peptides may determine the rate and kinetics of Aβ oligomerization. The morphology of the Aβ assemblies formed may also vary depending on the ratio of the isoforms, species, organ type and the biological properties of the surrounding cells^51, 52^. The exact interaction that occurs between the two peptides is not clearly known and may differ largely depending on above mentioned factors which in turn will determine their pathogenic effects. However, further studies are required to determine the molecular mechanism underlying this effect.

Although, the drusen-like deposits formed are immune-positive for Aβ, they were negative for Thioflavin S-a conventional amyloid dye. An earlier study has reported that drusen associated with AMD is immuno-positive for thioflavin^28^. It is possible that with age these drusen-like deposits will also become positive for thioflavin-S. However, further studies to examine the difference between thioflavin-S positive and negative Aβ aggregates and its implications in AMD progression are required.

Our present mice model develops multiple pathological features of dry AMD however, there are few inherent limitations of this model. Firstly, rodents do not have a macula/fovea and thus cannot mimic the human AMD condition completely. Secondly, while the drusen-like deposits in mice are located sub-retinal, in humans the drusen are formed sub-RPE (between the RPE and Bruch’s membrane). This difference in the location of drusen could be due to anatomical and physiological differences between rodent and human eye. A possible explanation being that lipid transport across RPE, which is important for drusen formation, is different in rodents and humans with lysosomal bodies (that bring about lipid transport across membranes) accumulating in the apical part of RPE in rodents and the basal part in humans^53^. Nevertheless, this model is valuable, to further explore the mechanistic aspects of Aβ peptides toxicities, proteostasis dysfunction in AMD, and to develop and assess disease-modifying therapies.

In conclusion, this study provides evidence to support the development of a novel time-accelerated mouse model for dry AMD by increasing the expression level of secreted Aβ in the outer retina. This model should be useful in understanding the underlying inter-relationship between Aβ build-up and retinal degenerative diseases, also to study the role of other risk factors like oxidative stress, inflammation and environmental factors such as high fat/high cholesterol diet in disease progression. Utilizing different serotypes of the AAV vector allows targeting of specific retinal cell types enabling us to study the effect of different species, titers and ratios of specific Aβ peptide combinations on specific retinal neurons. The availability of non-invasive *In vivo* methods to image the retina will allow us to follow the progression of disease and efficacy of different treatments without having to sacrifice the animals thus allowing us to do more long-term studies in the future. This should prove to be highly beneficial in developing novel therapies for treatment of AMD and to understand the molecular and cellular mechanisms underlying the toxic effects of Aβ peptides on specific retinal neurons.

## Methods

### Animals

Adult wild-type C57Bl/6 J mice were purchased from the Jackson Laboratory (Bar Harbor, ME). Two months old mice were used for this study and were kept at Animal Care Service at University of Florida. Animals were maintained under standardized conditions with an artificial 12 h–12 h dark-light cycle, with free access to food and water. All animal studies were performed in accordance with the Association for Research in Vision and Ophthalmology (ARVO) statement for the use of animals in Ophthalmic and Vision Research and were approved by the Institutional Animal Care and Use committee (IACUC) at the University of Florida.

### Subretinal Injection

Topical application of 2.5% Phenylephrine hydrochloride and 1% Atropine sulfate ophthalmic solutions were used to dilate the mouse eyes. Mice were anaesthetized by intra-peritoneal (IP) injection with Ketamine/Xylazine mixture (60 mg/kg and 10 mg/kg respectively), Eyes were punctured with a 30-gauge needle between the corneoscleral junction and the ora serrata into the vitreous cavity. Viral vector was injected to the subretinal region using a 33 gauge, 1.5-inch blunt needle attached to a 10 μl Hamilton syringe (Hamilton Company, Reno, NV) under a dissecting microscope. A volume of 1 μl of AAV1-BRI-Aβ42, AAV1-BRI-Aβ40, combination of AAV1-BRI-Aβ40+AAV1-BRI-Aβ42, or control vector AAV1-BRI viral particles [1 × 10^12^ vector genome(vg)/ml] was injected into each eye.

### Total Amyloid beta ELISA

To confirm the increase in level of total Aβ protein expression in injected eyes, retinal tissues were collected from each experimental group 8 week after subretinal injection and ELISA assays were performed. The retinal tissues were homogenized in Diethyl Amine (DEA) buffer using a hand held disposable micro tissue grinder (Pellet Pestle, Fisher Scientific, Waltham, MA). The DEA buffer contained 0.2% DEA in 50 mM NaCl solution and Complete Protease Inhibitor (Roche Applied Science, Indianapolis, IN). Homogenized tissue was centrifuged at 100,000 g for 30 minutes and supernatant collected. The supernatant was neutralized with 10% (v/v) 0.5 M Tris-HCl (pH = 6.8) immediately. Total Aβ levels were determined from the neutralized samples using a sandwich ELISA strategy: Aβ capture with mAb5 (human Aβ 1–5, T.E. Golde) and detection with HRP-conjugated 4G8 (human Aβ17–24; Covance, Princeton, NJ). The colorimetric reaction was developed with 3,3′,5,5′-Tetramethylbenzidine substrate and halted with 6.7% o-Phosphoric Acid (Fisher Scientific, Waltham, MA). The plates were analyzed using Molecular Devices spectrometer and Softmax Pro 6 software (Molecular Devices, Sunnyvale, CA). All measurements were performed in duplicate and the data represents the mean of at least two assay results.

### Fundus and Optical Coherence Tomography (OCT) imaging

A Micron III digital fundus retinal imaging microscope (Phoenix Research Laboratories, Pleasanton, CA) and a Bioptigen (Morrisville, NC) high resolution instrument for spectral domain optical coherence tomography (SD-OCT) was used to monitor retinal morphology and development of drusen like deposits every 2 weeks after sub-retinal injection of AAV1 vector encoding BRI-Aβ peptides. Mouse eyes were dilated same way as for subretinal injection. Mice were, then anesthetized with IP injection of a mixture of ketamine/xylazine as described above. To avoid loss of moisture from the ocular surface during the procedure, mice received a drop of 2.5% hypromellose ophthalmic demulcent solution (GONAK; AKORN, Lake Forest, IL). Bright-field fundus images and SD-OCT images were, acquired using the same exposure times for all eyes.

### Electroretinography

Mice were anesthetized by IP injection of Ketamine/Xylazine mixture and eyes were dilated as described above. Anesthetized mice were kept on a heating pad at 37 °C throughout the recordings. To avoid loss of moisture from the ocular surface during the procedure, mice received a drop of 2.5% hypromellose ophthalmic demulcent solution (GONAK; AKORN, Lake Forest, IL). A monopolar contact loop was placed on the surface of the cornea as the active electrode; needle electrodes under the scalp and in the tail served as reference and ground respectively. Full-field electroretinogram was recorded from both eyes following the International Society for Clinical Electrophysiology of Vision (ISCEV) standard protocol adapted for mice. For full-field scotopic ERG recordings, a series of increasing flash intensities (−2.7, −0.7, 0.3, 1.3, 2.3, and 2.6 log cds/m^2^) was presented to mice after overnight dark adaptation. Photoreceptor responses (a-waves) were elicited with flashes (0.02–2 cds/m^2^) and RPE- driven C-wave responses were elicited by a 2.6 log cds/m^2^ intensity flash and recorded within a period of 15 s after the flash presentation signals were amplified and averaged on a PC-based recording system.

### Histological analysis

Eyes were collected from control AAV1-BRI, AAV1-BRI-Aβ40, AAV1-BRI-Aβ42 and a combination of AAV1-BRI-Aβ40+AAV1-BRI-Aβ42 vector injected mice at 8 weeks after sub-retinal injection as well as from age-matched un-injected control mice and fixed in 4% paraformaldehyde followed by paraffin embedding. Paraffin sections (4 μm) were cut, deparaffinized and H&E staining was performed to evaluate the retinal morphology. Adjacent sections were stained with 4% thioflavin-S for 10 minutes. The images were captured on a light microscope using a 10X, 20X and/or a 40X objective lens. The number of drusen-like sub-retinal deposits were counted in a masked fashion from these images. The area, diameter and perimeter of drusen-like deposits were quantified using the ImageJ/NIH image analysis system.

### Immunostaining

For immunofluorescence studies, eyes were collected from each experimental group at 8 weeks after sub-retinal injection. The mice were first anaesthetized with ketamine/Xylazine mix as described above and then perfused transcardially with 4% paraformaldehyde freshly made in PBS (pH 7.4). Following perfusion, the eyes were, enucleated and post-fixed in 4% paraformaldehyde overnight at 4 °C. The eyes were then either processed and embedded in paraffin or cryoprotected in 30% sucrose in PBS at 4 °C and frozen in OCT compound (Tissue-Tek; Sakura-Finetek, Torrance, CA). 4 µm thick paraffin sections or 12 µm thick cryostat sections were, cut and mounted on Superfrost Plus slides. Paraffin sections were deparaffinised, rehydrated and subjected to antigen retrieval by boiling for 20 min in 10 mM sodium citrate pH 6.0 solution. Both paraffin and cryo sections were then incubated in blocking solution (5% BSA+0.3% Triton X100 in PBS) for 1 hour. This was followed by incubation with specific primary antibodies: mouse anti-pan Amyloid Beta (1:1000, T.E. Golde), mAb5 (human Aβ 1–5, T.E. Golde), rabbit anti-Iba-1 (1:200, DAKO, Carpinteria, CA), rabbit anti-C5b9 (1:500, Abcam ab21635,Cambridge, United Kingdom) and mouse anti-glia fibrillary acidic protein (GFAP) (1:500, Sigma, St Louis, MO) diluted in the same blocking solution (overnight at 4 °C) then with the appropriate secondary antibodies conjugated to Alexa 488 or 594 (Molecular Probes/Invitrogen, Eugene, OR) for 1 hour at RT. Sections were washed in PBS containing the nuclear counterstain DAPI (4′,6 diamidino-2- phenylindole), and mounted in Dako mounting media. For TUNEL staining the *In Situ* Cell Death Detection Kit, TMR red (Roche Applied Science, Indianapolis, IN) was used on paraffin sections in accordance to the manufacturer’s instructions. The images were captured at equal camera exposures (for each antibody staining) on a Keyence confocal microscope (Keyence Corporation of America, Ithasca, IL) or a spinning disc confocal (Ultra VIEW Vox, Perkin Elmer, Waltham, MA) using a 10X, 20X and/or a 40x objective lens and were prepared for presentation by making similar adjustments to brightness and contrast using Adobe Photoshop (Adobe Systems, San Jose, CA).

### Retinal RNA preparation, cDNA synthesis and qRT-PCR

Total RNA was extracted from freshly dissected adult mouse retinas using Trizol Reagent (Invitrogen, Carlsbad, CA) according to manufacturer’s instruction. Purified RNA was resuspended in 10 μl RNase-free dH2O. Reverse transcription was performed using Enhanced Avian HS RT-PCR kit (Sigma, St Louis, MO) following manufacturer’s instructions. Real time PCR was carried out on a real time thermal cycler (iCycler, Bio-Rad Life Sciences) using iQTM Syber Green Supermix (Bio-Rad Life Sciences, Hercules, CA). The threshold cycle number (Ct) for real-time PCR was set by the cycler software. Optimal primer concentration for PCR was determined separately for each primer pair. Each reaction was run in duplicate or in triplicate, and reaction tubes with target primers and those with β-actin primers were always included in the same PCR run. The expression levels of the different experimental genes were established based on the Ct compared with 2 different control housekeeping genes β-actin and GAPDH in each sample (amount of target = 2^−∆∆Ct^) and presented as fold change. Primer sequences used in this study are shown in Table 1. All the reactions were repeated at least twice.

**Table 1:** Primers Used for real-time RT-PCR.

Gene Name	Accession Number	Sequences
Interleukin-6	NM_031168.1	Forward: 5′-TCGGCAAACCTAGTGCGTTA-3′ Reverse: 5′-CCAAGAAACCATCTGGCTAGG-3′
IL-1β	NM_008361.3	Forward: 5′-AAAGCCTCGTGCTGTCGGACC-3′ Reverse: 5′-CAGCTGCAGGGTGGGTGTGC-3′
TNF-α	NM_013693.2	Forward: 5′-AGGCGCCACATCTCCCTCCA-3′ Reverse: 5′-CGGTGTGGGTGAGGAGCACG-3′
ICAM-1	NM_010493	Forward: 5′-AGATGACCTGCAGACGGAAG-3′ Reverse: 5′-GGCTGAGGGTAAATGCTGTC-3′
MCP-1	NM_011333	Forward: 5′-CCCCACTCACCTGCTGCTACT-3′ Reverse: 5′-GGCATCACAGTCCGAGTCACA-3′
VEGF	NM_031836	Forward:5′-TGCACCCACGACAGAAGGGGA-3′ Reverse:5′-TCACCGCCTTGGCTTGTCACAT-3′
β-Actin	X03672	Forward: 5′-AGCAGATGTGGATCAGCAAG-3′ Reverse: 5′-ACAGAAGCAATGCTGTCACC-3′
GAPDH	XM_017321385.1	Forward: 5′-TCCCAACTCGGCCCCCAACA-3′ Reverse: 5′-GGCTCCCTAGGCCCCTCCTG-3′

### Statistical analysis

When two individual experimental groups were analyzed, statistical analyses were performed using unpaired two-tailed Student’s *t*-tests. We used paired two-tailed Student’s *t*-tests for analyses comparing *AAV1-Bri-A*β*42, AAV1-Bri-A*β*40* and *AAV1-Bri-A*β*42*+*AAV1-Bri-A*β*40* injected retinas to un-injected and AAV1-BRI injected controls. Statistical significance was considered at *P* ≤ 0.05.

## Electronic supplementary material

Supplementary Information
