# Supplementary material for: Amyloid β peptides overexpression in retinal pigment epithelial cells via AAV-mediated gene transfer mimics AMD-like pathology in mice
Source: Sci Rep. 2017 Jun 12;7:3222. doi: 10.1038/s41598-017-03397-2 (PMC5468329; doi:10.1038/s41598-017-03397-2)
Supplement: Supplementary file 1 — Supplementary Information [file 41598_2017_3397_MOESM1_ESM.pdf]

Supplementary Data:

Amyloid β peptides overexpression in retinal pigment epithelial cells via AAV-mediated gene transfer mimics AMD-like pathology in mice

Tuhina Prasad[1], Ping Zhu[1], Amrisha Verma[1], Paramita Chakrabarty[2], Awilda M Rosario[2], Todd E Golde[2] and Qiuhong Li[1*]

1 Department of Ophthalmology, University of Florida, Gainesville, Florida, 32610, USA

2 Department of Neuroscience, Center for Translational Research in Neurodegenerative disease and McKnight Brain Institute, University of Florida, Gainesville, Florida, 32610, USA

*** Corresponding Author:**

Qiuhong Li, Ph.D.

Department of Ophthalmology

University of Florida,

Gainesville, Florida 32610-0284, USA

E-mail: qli@ufl.edu

Telephone: 352-392-0747

Fax: 352-392-0573

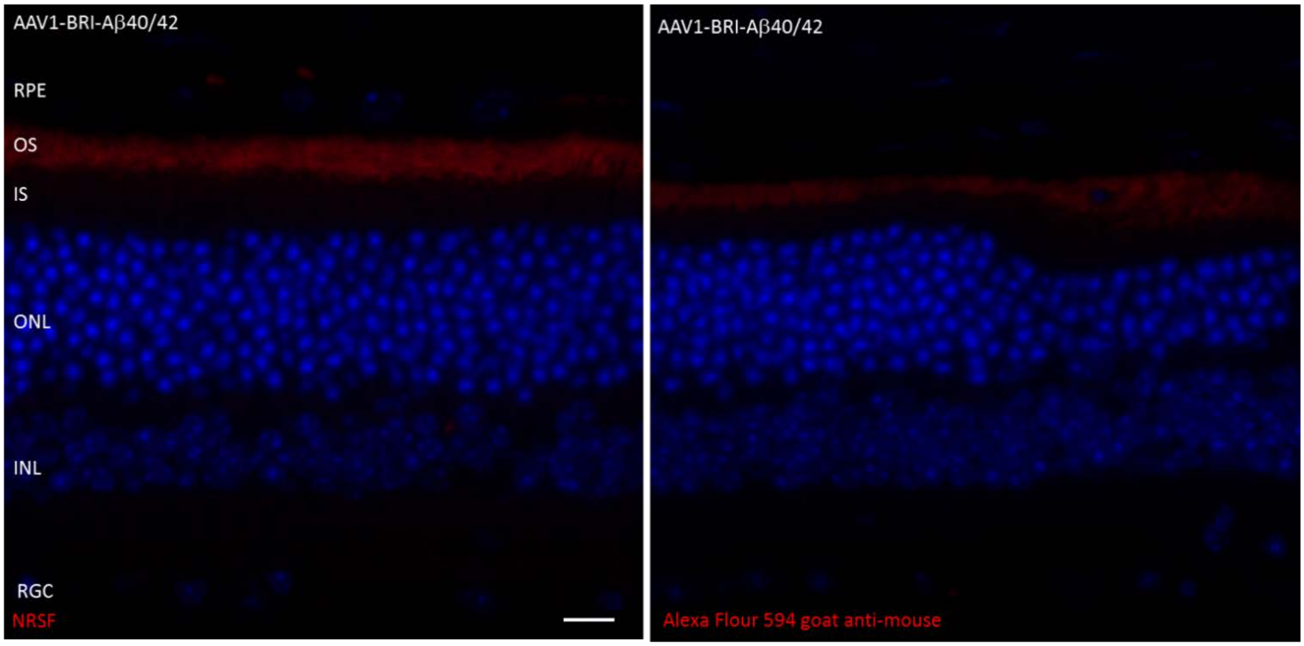

Supplementary Figure S1: Secondary only and Isotype control for pan Aβ staining

PFA fixed, paraffin embedded mouse retinas injected with AAV1-BRI-Aβ40/42 were stained with either a secondary antibody- Alexa Flour 594 goat anti-mouse alone at 1:500 dilution or a mouse monoclonal anti-NRSF antibody (sc-374611) at 1:1000 dilution (similar to that of pan Aβ antibody) as an isotype control followed by the appropriate secondary antibody. A similar faint pattern of staining is observed in both cases in the outer segment/RPE layer of the retina. Scale Bar = 20 μm. RPE: retinal pigment epithelium; OS: outer segment of photoreceptor; IS: Inner segment of photoreceptor; ONL: outer nuclear layer; INL: inner nuclear layer; IPL: inner plexiform layer; RGC: retinal ganglion cells.

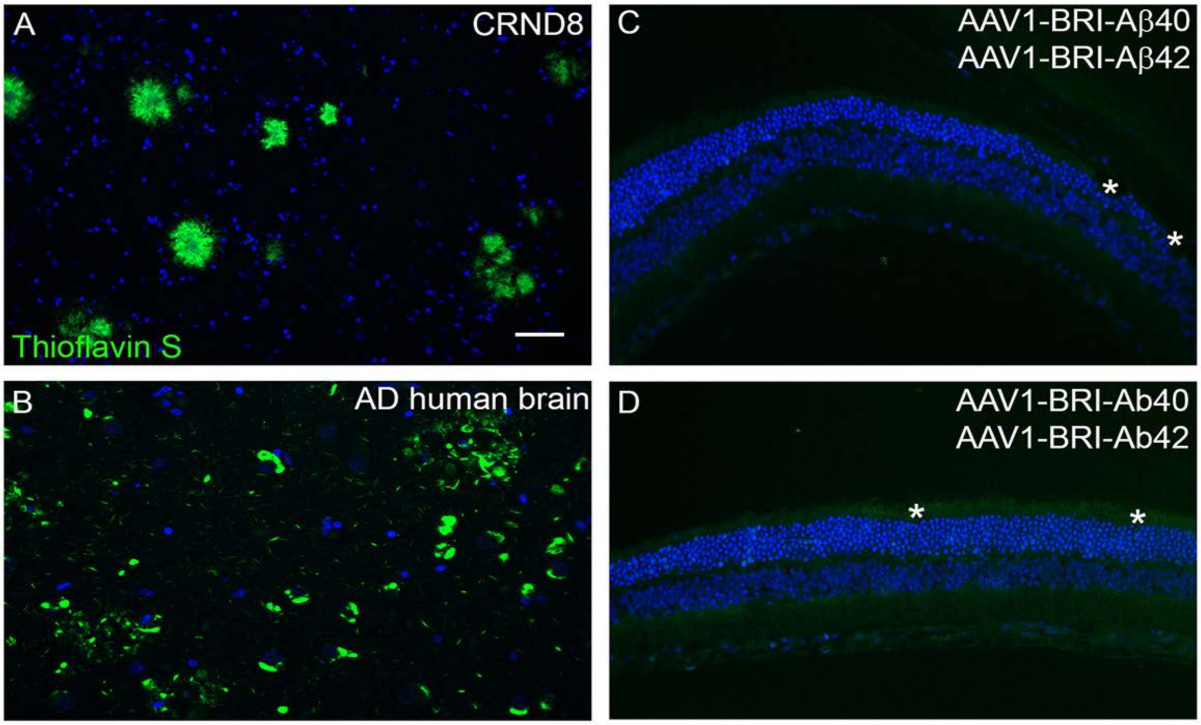

Supplementary Figure S2: Representative images of Thioflavin-S staining

Thioflavin-S immuno-positive amyloid deposits seen in paraffin embedded brain sections in the cortical region from (A) 20 months old transgenic CRND8 [55] mice and (B) human with Alzheimer's Disease. (C-D) Absence of thioflavin-S positive staining at drusen-like deposits (*) in paraffin embedded retinal sections from AAV1-BRI-Aβ40+AAV1-BRI-Aβ42 injected 4 months old C57BL/6J mice. Scale Bar: (A-D) 20μm.

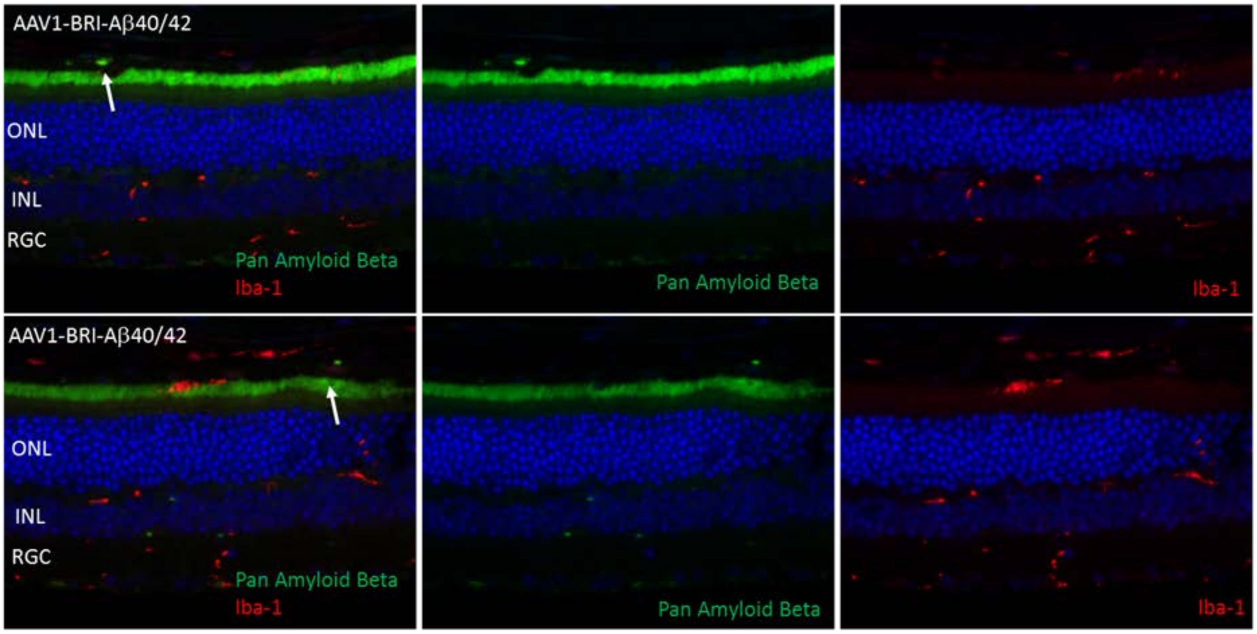

Supplementary Figure S3: Co-localization of pan Aβ and Iba-1 staining

PFA fixed, paraffin embedded mouse retinas injected with AAV1-BRI-Aβ40 and AAV1-BRI-Aβ42 were double immuno-stained with pan-Aβ and Iba-1 to assess co-localization of the two signals. Although activated microglia are present near the Aβ positive drusen-like deposits no co-localization of the Aβ and Iba1 signal is observed suggesting that there is no cross-reactivity of the secondary antibody with microglial Fc receptors. Scale Bar = 20 μm.ONL: outer nuclear layer; INL: inner nuclear layer; IPL: inner plexiform layer; RGC: retinal ganglion cells

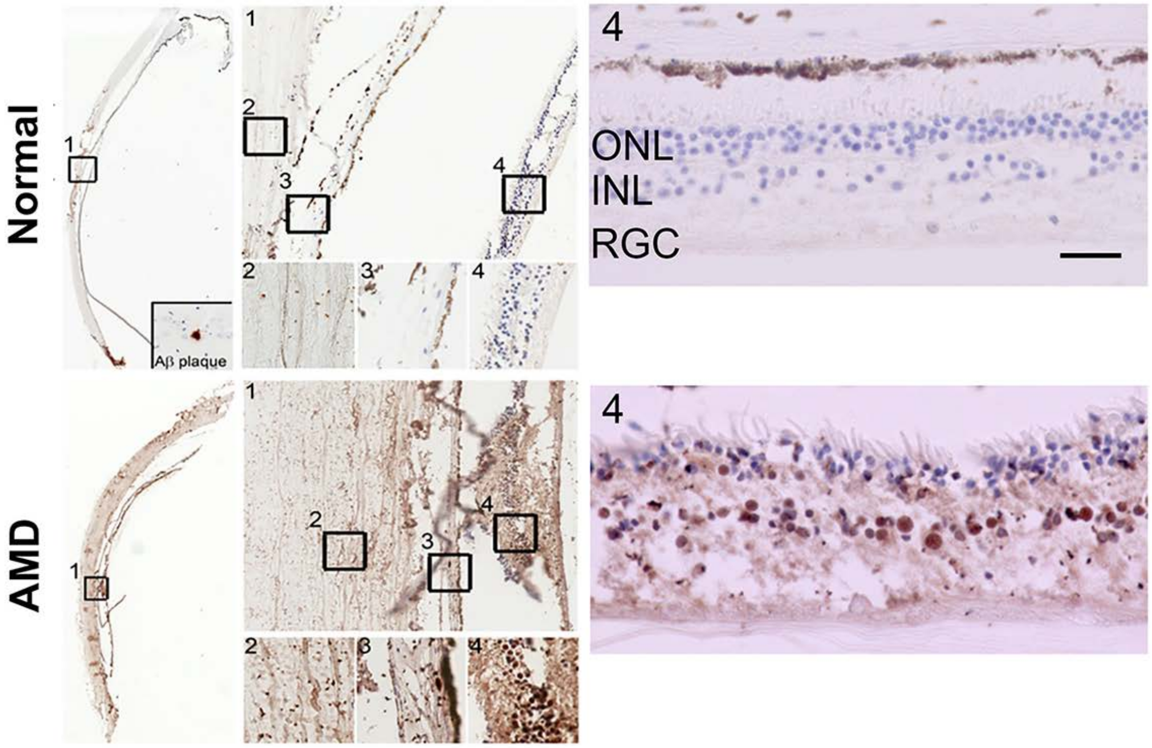

Supplementary Figure S4: Representative pan Aβ immunostaining of human retina.

Paraffin embedded human retinas were stained with biotin conjugated Ab5 (anti Aβ 1-5). Top panel is retina from a normal 88 years old human male while the lower panel depicts retina from an 85 years old male AMD patient. Left panel depicts a whole mount and panels on the right depict magnified images corresponding to selected areas of interest (numbered boxes). Box #1: Cross-section of posterior eye cup, Box #2: Choroid, Box #3: RPE, Box # 4 Retina. Scale bar: 20 μm. ONL: outer nuclear layer; INL: inner nuclear layer; IPL: inner plexiform layer; RGC: retinal ganglion cells. A representative Aβ plaque from a transgenic APP mouse stained with biotin conjugated Ab5 is shown as an inset in top left panel. Adult human donor eyes with and without AMD were procured from the Lions Eye Institute for transplant and research following informed consent (Tampa, Fl, USA).
